# The Gastric Phenotype in the Cypriniform Loaches: A Case of Reinvention?

**DOI:** 10.1371/journal.pone.0163696

**Published:** 2016-10-26

**Authors:** Odete Gonçalves, L. Filipe C. Castro, Adam J. Smolka, António Fontainhas, Jonathan M. Wilson

**Affiliations:** 1 Molecular Physiology Laboratory, CIIMAR, Porto, Portugal; 2 Institute of Ciências Biomédicas Abel Salazar, Universidade do Porto, Porto, Portugal; 3 Animal Genetics and Evolution, CIIMAR, Porto, Portugal; 4 Faculdade de Ciências, Universidade do Porto, Porto, Portugal; 5 Medical University of South Carolina, Charleston, South Carolina, United States of America; 6 University of Trás-os-Montes and Alto Douro, Vila Real, Portugal; 7 Wilfrid Laurier University, Waterloo, Canada; James Cook University, AUSTRALIA

## Abstract

The stomach, which is characterized by acid peptic digestion in vertebrates, has been lost secondarily multiple times in the evolution of the teleost fishes. The Cypriniformes are largely seen as an agastric order; however, within the superfamily Cobitoidea, the closely related sister groups Nemacheilidae and Balitoridae have been identified as gastric families. The presence of these most recently diverged gastric families in an otherwise agastric clade indicates that either multiple (>2–3) loss events occurred with the Cyprinidae, Catostomidae and Cobitidae, or that gastric reinvention arose in a recent ancestor of the Nemacheilidae/Balitoridae sister clade. In the present study, the foregut regions of Cobitidae, Nemacheilidae/Balitoridae and the ancestral Botiidae family members were examined for the presence of gastric glands and gastric proton pump (Atp4a) α subunit expression by histology and immunohistochemistry respectively. *Atp4a* gene expression was assessed by reverse transcriptase-polymerase chain reaction (RT-PCR). Gastric glands expressing apical H^+^/K^+^-ATPase α subunit and isolated partial sequences of *atp4a*, identified using degenerate primers showing clear orthology to other vertebrate *atp4a* sequences, were detected in representative species from Nemacheilidae/ Balitoridae and Botiidae, but not Cobitidae (*Misgurnus anguillicaudatus*). In summary, we provide evidence for an uninterrupted gastric evolutionary lineage in the Cobitoidea, making it highly improbable that the stomach was reinvented in the Nemacheilidae/Balitoridae clade consistent with Dollo’s principle. These results also indicate that the gastric trait may be present elsewhere in the Cobitoidea.

## Introduction

The acid-secreting stomach, the most highly diversified region of the gut, is a significant vertebrate innovation that probably emerged in the ancestor of gnathostomes about 450 MYA [1, 2, 3, 4]. Its success has been inferred from the conservation of acid-peptic digestion [4], which is intrinsically linked with gastric glands and the gastric proton pump. Thus, the secretion of HCl and aspartic protease pepsins into the gastric lumen define the vertebrate stomach [5]. The stomach is not merely an enlargement of the gut for food storage. In effect, it provides several selective advantages such as improved digestion through, acid-peptic digestion [3], acid lysis of plants cell walls and invertebrate exoskeletons [6], enhanced dietary calcium [4, 7] and phosphorus [8] uptake, and immune system defence by providing a chemical barrier to pathogen entry into the lower digestive tract [4, 9].

These advantages notwithstanding, secondary loss of the stomach (acid-peptic digestion) has occurred independently numerous times in vertebrate evolution [10, 11, 12], most noticeably in the teleost fishes (>15 times), which includes a recent estimate of 7% of families and 20–27% of species of fish [12]. Although numerous loss events have been documented, there are a number of clades in which the phylogeny of stomach loss remains controversial or unresolved due to uncertainties over phylogenetic relationships, and sparse and/or conflicting reports over the last 200 years, compounded by the sheer size and diversity of the teleost clade (28 000 species, [13]). The Cypriniformes present such an example, with Cuvier (1805) being the first to recognize the absence of a stomach in a cypriniform species.

Gastric acidification is achieved by ATP-catalyzed extrusion of H^+^ in exchange for K^+^ by the gastric proton pump. The gastric H^+^/K^+^-ATPase is a heterodimer composed of an α and β subunit encoded by the genes *atp4a* and *atp4b* with the stomach being the dominant site of expression [14]. The absence of the proton pump genes has been found to be correlated with the absence of gastric glands, the hallmark of the vertebrate stomach [12, 15]. Therefore the presence of these genes can serve as an indicator of gastric function.

**Fig 1:**
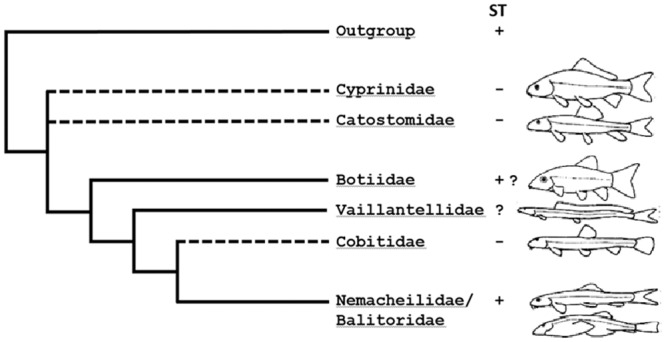
A simplified schematic of the phylogenetic relationships in the Cypriniformes (modified from Slechtova *et*. *al*. (2007) [22] and Wilson and Castro (2010) [12]). The dashed lines indicate families in which the absence of a stomach (-) is observed (Cobitidae and Cyprinidae/Catostomidae). The crown families Nemacheilidae/Balitoridae are known to be gastric (+). It is unknown if the stomachless condition exists in Villantellidae (?) and the presence of the stomach in Botiidae requires confirmation (+?) [12].

The Cypriniformes order, with six families, 321 genera and approximately 3268 species, is currently the largest monophyletic group of freshwater fishes and is native to Asia, Europe, Africa and North America [13]. Cypriniformes are comprised of two superfamilies, the Cyprinidoidea (carps and minnows) and Cobitoidea (loaches and suckers) (Fig 1). They are culturally, economically and scientifically important due to their diversity in morphology, ecology, physiology and distribution [16]. From an evolutionary perspective, Cypriniformes may provide insight into stomach evolution and loss since they are predominantly agastric through secondary loss. The Cyprinidae family is probably entirely agastric (e.g. [17, 18]) and within the superfamily Cobitoidea, the family Cobitidae has many agastric members (*Misgurnus* and *Cobitis spp*. [17, 19, 20, 21]). However, unexpectedly, there is known to be at least one closely related pair of families, the Nemacheilidae and Balitoridae (Fig 1; [22, 23, 24]) in which there is histological evidence for gastric glands (*Nemacheilus angorae* [25], *Barbatula barbatula* [26], *Nemacheilus brandti* [27] and *Lefua costata* [27]). The presence of these apical group gastric families in an otherwise agastric clade indicates that either multiple (>2–3) loss events could have occurred or gives rise to the possibility of a secondary reinvention of the stomach in the Nemacheilidae and Balitoridae families. This latter possibility would contradict a concept in evolutionary biology known as Dollo’s principle, which states that lost complex characters are not regained and characters cannot re-evolve because the genetic and/or developmental features underlying an unexpressed character accumulate mutations that are highly unlikely to be reversed [28]. Given the close relationship between stomach loss and gastric gene loss, evolutionary reacquisition of a stomach affords a unique opportunity to test Dollo’s principle.

In order to study a gastric lineage in the Cypriniformes we examined representative species from the family Botiidae, which is ancestral to both Cobitidae and Nemacheilidae/Balitoridae clades [22]. It should be noted that Verigina [27] reported the presence of gastric glands in *Botia hymenophisa* (family Botiidae) but no images were provided. In addition, in some gobies (family Gobiidae, order Perciform) where stomach loss has been reported [12], glandular structures were claimed to be non-functional as acid-peptic gastric glands because of their poor development and mucopolysacharide content [29, 30]. This, and the absence of a protective mucus-secreting epithelium lining the stomach, suggested functionality as mucous secreting glands. To overcome this uncertainty, the present study documented the presence of gastric glands by histology, and gastric proton pump α subunit (HKα1 = Atp4a /*atp4a*) expression by immunofluorescence microscopy and RT-PCR. The presence of a stomach in this family would indicate that stomach loss occurred in the Cobitidae rather than stomach reinvention in the Nemacheilidae/Balitoridae. In our earlier work [12] we have presented some preliminary data on gastric glands histology and Atp4a immunohistochemistry in Botiidae which we have expanded in the present study and complemented with genetic data in a number of additional species from the families Botiidae, Nemacheilidae, Balitoridae and Cobitidae.

## Materials and Methods

### Animals

Clown loach (*Chromobotia macracanthus*), golden zebra loach (*Botia histrionica*), hillstream loach (*Beaufortia kweichowensis*), and Asian weatherloach (*Misgurnus anguillicaudatus*) were obtained from aquarium fish suppliers and kept on a natural photoperiod in glass aquaria with filtered, dechlorinated tap water. Angora loach (*Nemacheilus angorae*) were obtained from Iran as field-collected specimens fixed in formalin (S. Malakpoor, Gorgan University of Agricultural Sciences and Natural Resources, College of Fisheries and Environment, Gorgan, Iran). Iberian loaches (*Cobitis paludica*) were collected locally (Mondego River, Portugal) at an earlier date as archival formalin-fixed, paraffin-embedded (FFPE) samples. Fresh tissue for RNA extraction was not available (protected status: Appendix III of the Bern Convention). Consequently the latter two species were analyzed by histology and immunohistochemistry only. Channel catfish (*Ictalurus punctatus*) and Mexican tetra (*Astyanax mexicanus*) were also collected and fixed for use as out group controls for IHC. Animals were treated in accordance with the Portuguese Animal Welfare Law (Decreto-Lei no.197/96) and animal protocols approved by the Centro de Investigação Marinha e Ambeintal (Universidade do Porto) and the Direção-Geral de Veterinária (Ministry of Agriculture).

### Sampling

Loaches were anaesthetized [1:5000 MS-222 (Pharmaq, UK), pH 7.5 adjusted with NaHCO_3_] and killed by cervical transection. In one set of animals (*n* = 3) the gastrointestinal tract was flushed *in situ* with RNAlater (Ambion, Austin USA), excised and stored according to the manufacturer’s instructions (24h at 4°C, then at -20°C). In a separate set of animals (*n* = 3), the gastrointestinal tract was flushed with 10% neutral buffered formalin (3.7% Formaldehyde/ PBS, pH = 7.3), the body cavity was opened by a ventral incision, and the entire fish was immersion-fixed for 24h at 4°C.

### Histology and Immunochemistry

Following fixation, tissues were dehydrated through an ethanol series, cleared with Clear Rite (Richard Allen Scientific, Kalamazoo MI) and embedded in paraffin (Type 6; Richard Allen Scientific). Sections were cut at 5μm with a Reichert Biocut 2030 microtome and stained with hematoxylin-eosin, Alcian blue (pH 2.5) and/or Periodic Acid Schiff staining protocols. Serial gastrointestinal tract regions were imaged with a Leica DFC300FX digital colour camera mounted on a Leica DM 6000 B microscope. Images were imported into Photoshop CS2 to resize and adjust brightness and contrast while maintaining the integrity of the data.

For immunohistochemistry, paraffin sections (5μm) were collected onto APS (3-aminopropyltriethoxysilane; Sigma-Aldrich, St Louis, MO)-coated slides, air dried and dewaxed. Sections were rehydrated in TPBS (0.05% Tween-20 in phosphate buffered saline, pH 7.4 [TPBS]), blocked with 5% normal goat serum in 1% bovine serum albumin (BSA)/TPBS for 20 minutes at room temperature, and then isolated using a hydrophobic barrier (PAP pen, Sigma-Aldrich). Primary antibodies (see below 2.3.1 Antibodies) were diluted in BSA/TPBS and incubated overnight at 4°C in a humidified chamber. Negative control sections were incubated with normal rabbit serum (NRS), normal mouse serum (NMS) or BSA/TPBS alone. After several washes with TPBS over 30 minutes, the sections were incubated at 37°C with goat anti-mouse Alexa Fluor 568 and goat anti-rabbit Alexa Fluor 488 conjugated secondary antibodies, diluted 1:400 (Invitrogen, Carlsbad, USA) in BSA/TPBS for 1h. Nuclei were stained with DAPI (4´, 6-diamidino-2-phenylindole, Sigma- Aldrich) during the second wash and coverslips were mounted with glycerol based fluorescence mounting medium (10% Mowiol, 40% glycerol, 0.1% 1, 4-diazabicyclo [2.2.2] octane (DABCO), 0.1 M Tris (pH 8.5). Imaging of sections was carried out as described above using a Leica DFC 340FX camera with the DM6000B microscope.

### Antibodies

The gastric H^+^/K^+^-ATPase α-subunit (HKα1 = Atp4a) was detected using the C2 rabbit polyclonal antibody raised against a synthetic peptide based on the 16 carboxyl-terminal amino acids of porcine gastric H^+^/K^+^-ATPase α-subunit [31]. The C2 antibody stains the apical cytoplasm of pig and rat gastric parietal cells as well as kidney collecting duct intercalated cells. The antibody has also been shown to be crossreactive with Atlantic stingray H^+^/K^+^-ATPase α-subunit in stomach and gill [32, 33]. Diluted neat serum was used in all experiments. In addition, an affinity-purified pan-specific P-type ATPase antibody was used [34]. This antibody (NAK121/αR1) was raised [35] [36] against a synthetic peptide sequence that is highly conserved between Na^+^/K^+^-ATPase (Atp1a), and both gastric (Atp4a) and non-gastric H^+^/K^+^-ATPase (Atp12a) α subunits (see S1 Fig). The NAK121 antibody has been used in a number of teleost studies to localize Na^+^/K^+^-ATPase α-subunit (see [37]).

Na^+^/K^+^-ATPase was specifically detected using the α5 mouse monoclonal antibody specific to the α-subunit (Atp1a) developed by Takeyasu et al [38]. This antibody has been used in a number of teleost studies [37], and was obtained as culture supernatant from Developmental Studies Hybridoma Bank, University of Iowa under contract N01- HD-7-3263 from NICHD.

Na^+^:K^+^:2Cl^-^ cotransporter (NKCC) was detected using the T4 mouse monoclonal antibody developed by Christian Lytle (University of California Riverside, CA; Lytle *et al*. 1995). This antibody has also been used to detect branchial NKCC in a number of different teleosts (see Wilson *et al*. [34]) and also shows crossreactivity with Na^+^:Cl^-^ cotransporter [39].

### Total RNA isolation and cDNA synthesis

Frozen stomach/anterior intestinal tract samples in RNAlater were weighed and blotted dry. Total RNA was isolated using the Aurum total RNA mini kit (BioRad, Hercules, CA USA) according to the manufacturer’s instructions. For *M*. *anguillicaudatus*, RNA was also isolated using either Qiagen RNeasy mini kit or GE minispin column kit. RNA concentration and purity were assessed by UV spectroscopy (Genova Jenway, Staffordshire, UK) at 260/280nm wavelength and integrity by electrophoresis (BioRad, Hercules CA USA) in a 1.2% formaldehyde agarose gel stained with Gel Red^™^ nucleic acid gel stain (Biotium, Hayward, CA, USA) and viewed with a LAS 4000 Mini Imager (FujiFilm, Tokyo, JP). The RNA samples were stored at -80°C.

The cDNA was synthesized from 1μg of total RNA in a 20 μl reaction volume using the iScript cDNA synthesis kit with the iScript reverse transcriptase (BioRad). *Misgurnus anguillicaudatus* RNA was also converted to cDNA using the Invitrogen Superscript III kit. The reactions were carried out according to manufacturers’ instructions using a BioRad MJ mini personal thermal cycler (BioRad) and samples stored at -20°C.

### PCR and gene isolation

Degenerate primers designed by Choe et al [33] from conserved regions of Atp4a in *Xenopus laevis* (GenBank accession number AAA76601) and *Pleuronectes americanus* (GenBank accession number AAD56285) were used to isolate *atp4a* genes from *C*. *macracanthus*, *B*. *histrionica* and *B*. *kweichowensis* (Table 1). In *M*. *anguillicaudatus*, these primers in addition to an expanded battery of degenerate and consensus primers, designed using iCodeHop [40] and Primer3 [41], were tested (S1 and S2 Tables). β-Actin primers (originally described by Santos et al [42]) for *Sparus aurata* were used for normalization of gene expression data.

**Table 1:** Summary of histology for the presence/absence of gastric glands and immunofluorescence (IF) microscopy results for HKα1 expression.

Family and Common Name		Species	Gastric glands	HKα1 IF
Botiidae	Clown loach	*Chromobotia macracanthus*	+	+
	Golden Zebra loach	*Botia histrionica*	+	+
Balitoridae	Hillstream loach	*Beaufortia kweichowensis*	+	+
Nemacheilidae	Angora loach	*Nemacheilus angorae*	+	+
Cobitidae	Iberian loach	*Cobitis paludica*	-	-
	Weather loach	*Misgurnus anguillicaudatus*	-	-

The *atp4a* PCR reactions were performed with 0.8 μl of sample cDNA and 0.5 μM of each primer in a 20μl reaction volume using Phusion Flash High-Fidelity PCR Master Mix (Finnzymes, Espoo, Finland). PCR conditions consisted of a denaturation step of 10 sec at 98°C, followed by 40 cycles of denaturation for 1s at 98°C, annealing for 5s at 54°C, and elongation for 15s at 72°C and a final elongation step for 1 min at 72°C.

Single bands in the predicted size range were retrieved from 2% agarose gels in TBE (Tris-borate-EDTA) buffer using GFX columns (GE Healthcare, Carnaxide, Portugal) and either directly sequenced (StabVida, Oeiras, Portugal) or cloned as follows. An a-tailing reaction was required for cloning and performed with 0.5 U of DyNAzyme II Polymerase, 0.2mM of dATP and 1x Finnzymes F-511 DyNAzyme buffer in 10μl reaction volume for 25 min at 72°C using a Duppio thermocycler (VWR Leuven, Belgium). Ligations and transformations of PCR products into JM109 high efficiency competent cells were performed with the pGEM^®^-T Easy vector systems kit (Promega Madison WI USA). Transformed cells were plated on Luria Bertani (LB) agar containing ampicillin (100 μg mL^-1^) and IPTG/X-gal for blue/white screening. Plates were incubated at 37°C overnight and transformants were selected, inoculated into 2 mL LB broth with ampicillin (100 μg mL^-1^), and incubated at 37°C overnight. Plasmid DNA was isolated using the Wizard^®^ *Plus* SV minipreps DNA purification system (Promega) and forward- and reverse-sequenced (Stabvida) with M13 primers [43].

β-Actin PCR was carried out with 0.8 μl of cDNA, 2mM MgCl_2_, 10mM dNTP and 0.8 U of DyNAzyme II Polymerase (Finnzymes) in an 20 μl reaction volume. The PCR profile included an initial denaturation step of 2 min at 94°C, followed by 30 cycles of denaturing, 30s at 94°C, annealing, 30s at 60°C and elongation, 30s at 72°C and a final elongation step of 5 minutes at 72°C.

PCR products were resolved in 2% agarose gel in TBE buffer run at 80V together with 100 bp DNA ladders (Bioron, Ludwigshafen, Germany) to confirm expected size of amplification products. Gel Red^™^-stained agarose gel images were acquired with a FujiFilm LAS-4000mini imager and imported into Photoshop CS2 to resize and adjust brightness and contrast while maintaining the integrity of the data.

Plasmids were initially edited for vector contamination using VecScreen, and insert sequence identities were initially confirmed by tBlastx search. Alignment and assembly was performed using BioEdit (Version 7.0.9.0; [44]) with ClustalW. Nucleotide sequences were translated using the translate tool of the ExPASy Proteomics server. Additional sequence data for the phylogenetic analysis were obtained from UniProt, GenBank and Ensembl. The GenBank or Ensembl accession numbers of the sequences used are as follows: ATP4A- *Siniperca chuatsi*: ADK25708.1 (GenBank); *Monodelphis domestica*: ENSMODP00000017413; *Oryctolagus cuniculus*: NP_001095171.1 (GenBank); *Anolis carolinensis*: ENSACAP00000010069; *Gasterosteus aculeatus*: ENSGACP00000011775; *Dasyatis sabina*: AAP35241.1 (GenBank); *Homo sapiens*: ENSP00000262623; *Mus musculus* ENSMUSP00000131964; *Ictalurus punctatus*: XP_017325785.1 (GenBank); ATP12A- *Homo sapiens*: NP_001172014.1(GenBank); *Mus musculus*: NP_619593.2 (GenBank); *Xenopus_laevis*: NP_001080818.1(GenBank); ATP1A(1)- *Gasterosteus aculeatus*: ENSGACP00000018907; *Ictalurus punctatus*: XP_017325785.1 (GenBank).

Amino acid sequence alignments were created with ClustalW in BioEdit. The evolutionary history was inferred by using the Maximum Likelihood method based on the JTT matrix-based model [45]. The evolutionary history was also inferred using the Neighbor-Joining method [46]. The tree was drawn to scale, with branch lengths measured in the number of substitutions per site. The distances were computed using the Poisson correction method [47] and are in the units of the number of amino acid substitutions per site. All positions containing gaps and missing data were eliminated. Evolutionary analyses were conducted in MEGA6 [48].

## Results

### Histology

**Fig 2:**
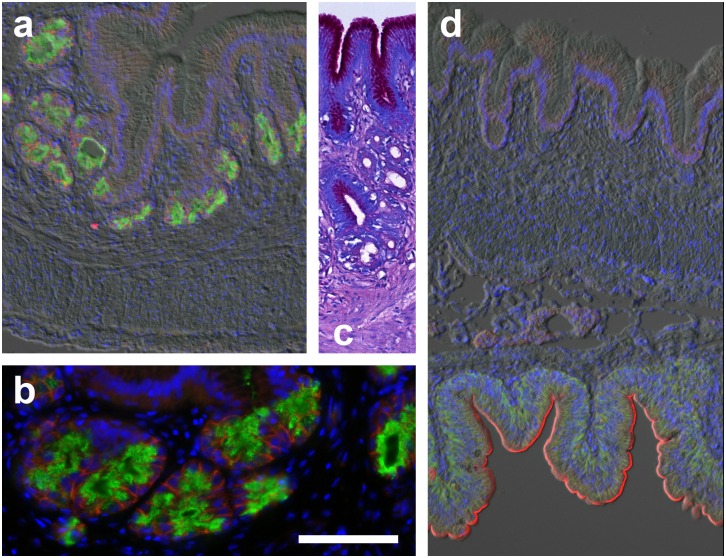
*Chromobotia macracanthus* (Botiidae) stomach and intestine. (a,b) Demonstration of acinar-type gastric glands expressing apical H^+^/K^+^-ATPase (HKα1; green) as determined by immunofluorescence microscopy in *Chromobotia macracanthus* (Botiidae). In the gastric glands Na^+^:K^+^:2Cl^-^ cotransporter (red) has a basolateral distribuition. c) PAS staining (magenta) indicates the presence of a protective mucous secreting epithelium in the stomach. (d; bottom) The intestinal epithelium shows the presence of typical intestinal enterocytes with basolateral Na^+^/K^+^-ATPase (green) and brush border Na^+^:K^+^:2Cl^-^ cotransporter (red) staining, while the pyloric region of the stomach (d; top) shows no such staining. Scale bar (a,c,d) 100 μm (b) 50 μm.

Gastric glands were observed in histological sections of foregut regions of loaches in the families Botiidae (*C*. *macracanthus*, *B*. *histrionica*) and Nemacheilidae/Balitoridae (*B*. *kweichowensis*, *N*. *angorae*) (Table 1). The structure of the gastric glands was variable between the species examined; however, the differences do not appear family dependent. *Beaufortia kweichowensis* has tubular glands that extend deep into the mucosa, while *B*. *histrionica* and *N*. *angorae* have shorter tubular glands and *C*. *macracanthus* has acinar-type glands. The gastric gland oxynticopeptic cells showed no PAS or AB staining, however, strong PAS staining was observed in the columnar epithelium lining the lumen of the stomach and the gland neck region of *C*. *macracanthus* (Fig 2). Similar PAS positive columnar mucocytes were observed in all of the gastric loaches. Goblet-type mucocytes were not observed in the stomach epithelium.

**Fig 3:**
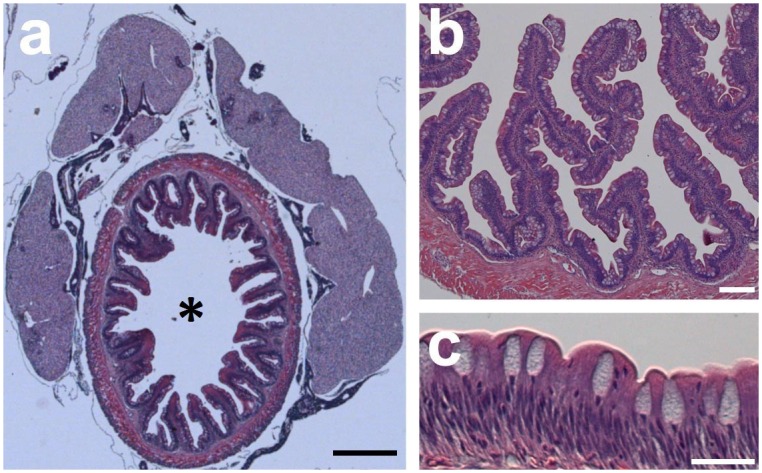
Histology of *Misgurnus anguillicaudatus* (Cobitinae) anterior intestine indicating the absence of gastric glands. The intestine is thrown into longitudinal folds and is covered by a columnar epithelium regularly interspersed with goblet cells. H&E staining viewed with differential interference contrast technique. Gut lumen (*). Scale bars (a) 500, (b) 100, (c) 25 μm.

Regarding the Cobitidae family, glandular tissue in the foregut region was not observed in either of the Cobitinae species (*M*. *anguillicaudatus*, or *C*. *paludica*). Instead the transition from the esophagus to the intestine is marked by a valve with a transitional columnar epithelium showing PAS staining. Distal to the esophagus is a typical intestinal lining with longitudinal folds covered by a columnar epithelium with a brush border (Fig 3). Mucous cells in this region are of the goblet type.

### Immunochemistry

**Fig 4:**
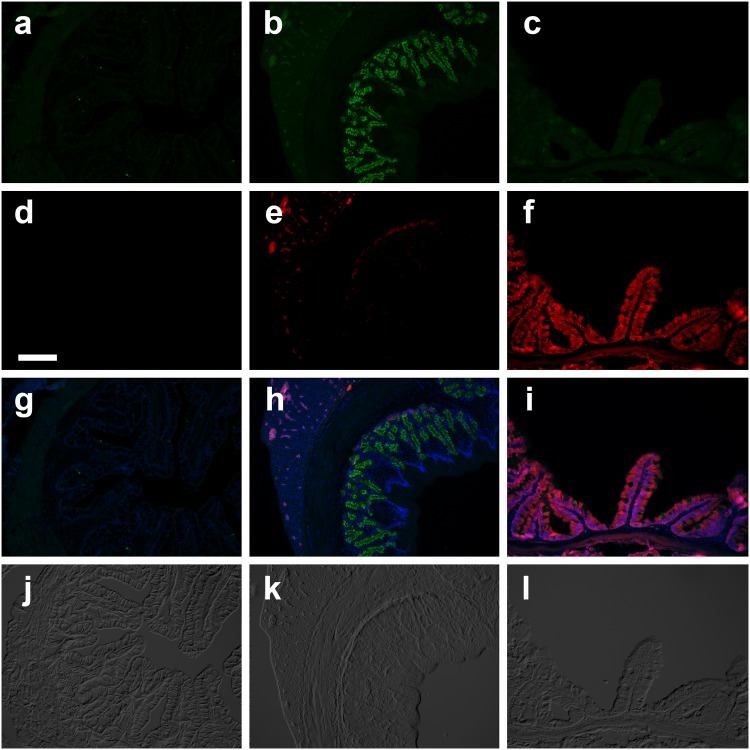
*Nemacheilus angorae* (Nemacheilidae) anterior gastrointestinal tract immunohistochemistry. Double immunofluoresent localization of HKα1 (**a,b,c**; green; C2 antibody) and NKAα1 (**d,e,f**; red; α5 antibody) in *N*. *angorae* esophagus (**a,d,g**), cardiac stomach (**b,e,h**) and proximal intestine (**c,f,i**). IHC images are overlaid with corresponding DAPI (**g,h,i**) and DIC (**j,k,l**). Scale bar 100 μm.

**Fig 5:**
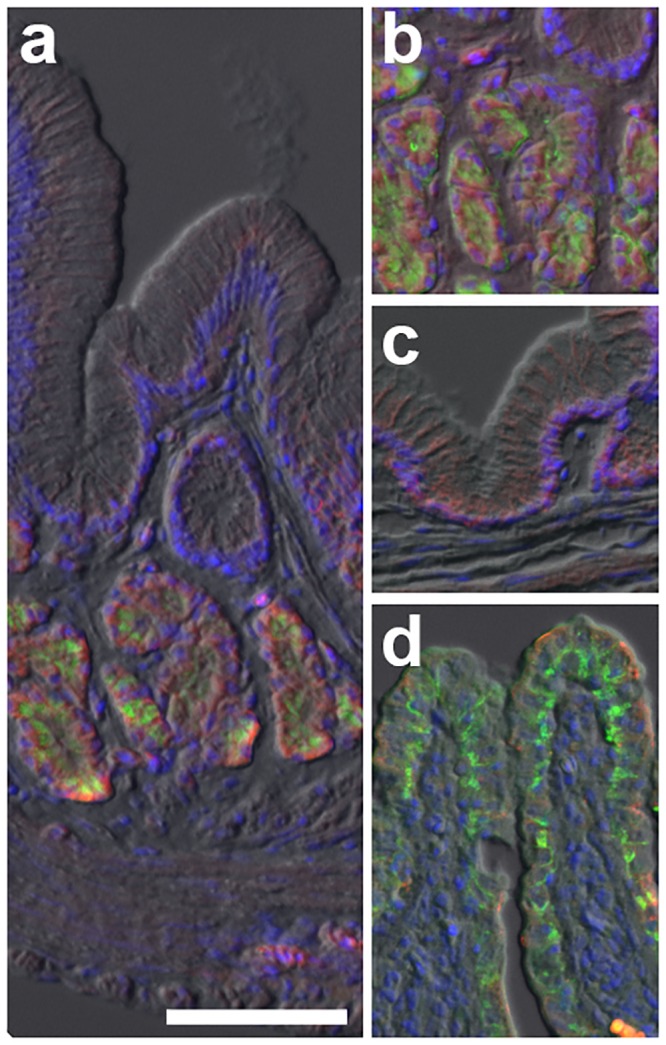
*Botia histrionica* (Botiidae) stomach and intestine immunohistochemistry. Immunohistochemical staining of golden zebra loach cardiac (a,b) and pyloric (c) stomach and intestine (d). Sections are probed with the HKα1/NKα1 antibody (αR1; green a,c,d) and HKα1 antibody (C2; green; b) and and colabeled with NKCC (T4; red) and NKα1 (α5; red) and respectively. Sections are counter stained with the nuclear stain DAPI (blue) and over laid with the DIC image for orientation. Scale bar 100μm.

Sections of stomach and anterior intestine of loach species were probed with HKα1 C2 antibody to detect expression of H^+^/K^+^-ATPase α subunit. Strong apical HKα1 immunoreactivity was localized to the gastric glands of Botiidae (*C*. *macracanthus*, *B*. *histrionica*), Nemacheilidae (*N*. *angorae*) and Balitoridae (*B*. *kweichowensis*,) (Table 1). Staining with the HKα1 C2 antibody showed strong apical immunoreactivity in the gastric glands of *N*. *angorae* (Fig 4), *B*. *kweichowensis*, (data not shown) and *B*. *histrionica* (Fig 5), and no immunoreactivity in esophagus, pyloric stomach and intestine. Similar labeling results were observed in *C*. *macracanthus* and *B*. *kweichowensis* with this antibody (not shown). In the outgroup Siluriformes channel catfish (*I*.*punctatus*) and Characiformes Mexican tetra (*A*. *mexicanus*) both expressed robust apical staining of HKα1 with the C2 antibody within their gastric glands (S2 Fig).

**Fig 6:**
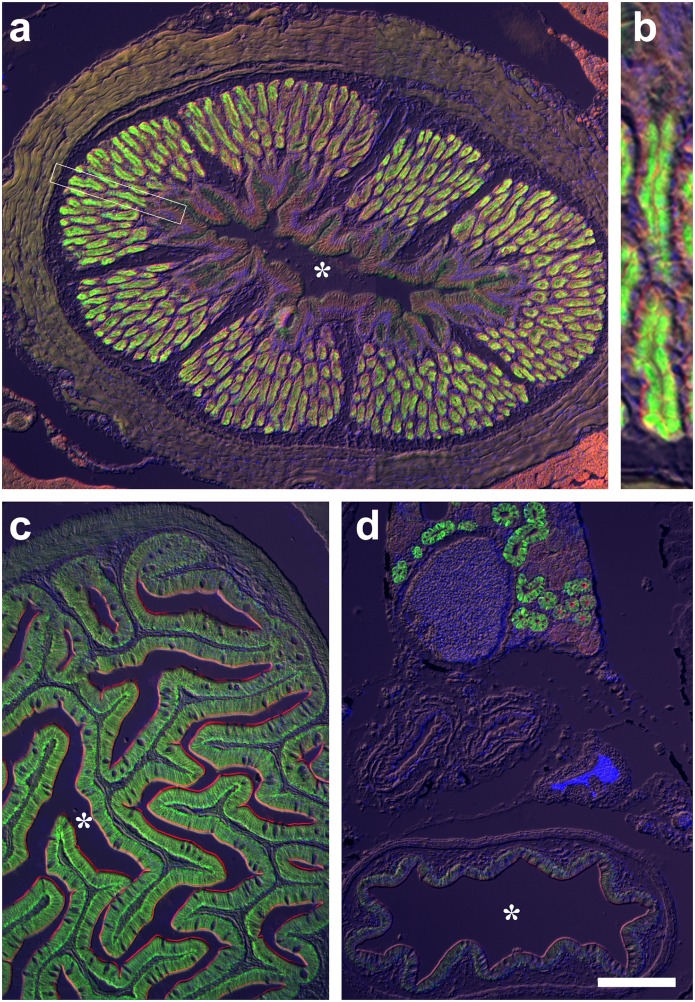
*Beaufortia kweichowensis* (Balitoridae) immunohistochemistry. Double immunohistochemical localization of HKα1/NKα1 (green; αR1 antibody) and NKCC (red; T4 antibody) in *B*. *kweichowensis* (**a,b**) cardiac stomach, (**c**) proximal intestine and (**d**) kidney (upper) and distal intestine (lower). Asterisk indicate gut lumen. Scale bar (a,c,d) 100 μm (b) 25 μm.

Complementary sections were also probed with a P-type ATPase IIc antibody originally developed to detect Na^+^/K^+^-ATPase subunit α1 (αR1; [34, 35, 36]). This antibody is predicted to crossreact with HKα1, HKα2 and NKAα based on conservation of the antigenic peptide sequence (S1 Fig). In gastric glands, staining with the αR1 antibody was apical (Figs 2a, 2b, 5a, 5b, 6a and 6b) in a pattern identical to the HKα1 antibody C2, and basolateral in the intestinal epithelium (Figs 2d, 5d and 6c), in a pattern identical to NKAα antibody α5 (Fig 4f). Conversely, basolateral staining in gastric gland oxynticopeptic cells could be demonstrated with the NKCC T4 antibody which crossreacts with the NKCC2 isoform, while in the adjacent intestinal epithelium, apical brush border immunoreactivity of the NKCC1 isoform was observed (Figs 2d and 5d). NKAα expression in the oxynticopeptic cells was generally very weak to non-detectable in contrast to HKα1 (Figs 4e, 5a and 5b).

### PCR and gene isolation

*Chromobotia macracanthus*, *B*. *histrionica* and *B*. *kweichowensis* cDNA PCRs using degenerate *atp4a* primers yielded two overlapping partial sequences within the predicted size range. These partial *atp4a* PCR products were cloned, sequenced, and aligned to obtain partial sequences of approximately 2.1 kb (GenBank accession number: JF934690.1, JF934691.1 and JF934689.1 respectively). Overall, the reported sequences cover a significant part of the expected size of the ORF of *atp4a*. Sequence alignments of putative gastric proton pump H^+^/K^+^-ATPase α subunits of others species indicate that they share a strong degree of identity in protein functional and other regions (S3 Fig), and are robustly grouped phylogenetically. In contrast, *M*. *anguillicaudatus* cDNA PCRs using the same degenerate primers and a set of additional *atp4a* degenerate and consensus primers (S2 Table) yielded either no reaction products, or bands in the predicted product size ranges that could not be confirmed as *atp4a* orthologues (non-specific amplification products). Touch-down, touch-up, and nested PCR protocols were also tested without success using alternative *M*. *anguillacaudatus* RNA and cDNA isolation protocols. The β-actin reference gene gave a single band with 250 bp in all samples.

### Phylogenetic analysis

The degree of amino acid identity between our partial loach sequences ranged from 88% to 93% and in comparison with the other teleosts ranged from 90% to 94%. The comparison with the cartilaginous fish, Atlantic stingray *Dasyatis sabina*, shows an identity of 89% with the *C*. *macracanthus*, *B*. *histrionica* and *B*. *kweichowensis* Atp4a’s. A comparison with representatives from other major vertebrate clades, shows that the percentage of amino acid identity ranges between an average of 89% in comparison with amphibians, 90% for reptiles and 86% for mammals. The closest phylogenetic P-type IIc ATPase to Atp4a is the Atp12a paralogue. The amino acid sequence identity to the Atp12a paralogue varies from an average of 72% (mammals) to 74% (amphibians). Curiously, Atp12a is so far not described outside tetrapods. The other related P-type IIc ATPase is Atp1a (Na^+^/K^+^-ATPase α subunit) that shows, on average, a degree of identity with our three sequences of less than 70%.

**Fig 7:**
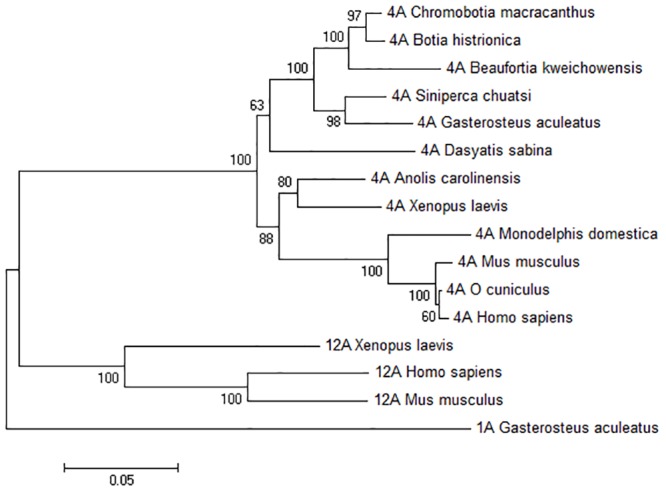
Molecular phylogenetic analysis of the three loach sequences. Phylogenetic tree obtained using the Maximum Likelihood method based on the JTT matrix-based model [45]. The percentage of trees in which the associated taxa clustered together is shown next to the branches.

A phylogenetic comparison of *C*. *macracanthus*, *B*. *histrionica* and *B*. *kweichowensis* partial Atp4a sequences with ATP4A, ATP12A and ATP1A1 sequences from representative taxa (Fig 7) reveals a high degree of homology with Atp4A sequences from other teleosts such as Chinese perch (*Siniperca chuatsi*), stickleback (*Gasterosteus aculeatus*) and channel catfish (*I*. *punctatus*).

## Discussion

The present study tested the hypothesis that gastric phenotype and proton pump expression are intrinsically linked [15]. Gastric histological, immunocytochemical, and molecular data acquired from loach species of the Nemacheilidae/Balitoridae and Cobitidae families correlated with the presence and absence of gastric glands with the presence and absence of proton pump *atp4a* gene expression. Further, the data established that members of the proximal ancestral family, the Botiidae, possess both gastric glands and *atp4a* expression thus providing clear evidence for a retention of a gastric lineage within the loaches.

### Stomach phenotype

The oesophagus and stomach are histologically distinguished by the transition from mucous goblet cells in esophageal epithelium to columnar mucous cells in the gastric epithelium, and more significantly, the presence of gastric glands [12]. Generally, multicellular glands are found only in the stomach in teleost fishes and not in other regions of the GI tract [12]. Histology clearly identifies gastric glands in both Botiidae species (*C*. *Macracanthus* and *B*. *histrionica*) and in both Nemacheilidae (*N*. *Angorae*) and Balitoridae (*B*. *kweischowensis*) species (Table 1). In contrast, no glandular tissue was observed in the foregut region of either Cobitidae species (*C*. *paludica* and *M*. *anguillicaudatus*).

Furthermore, we document the presence of *atp4a* orthologues and pronounced apical membrane localization of HKα1 antibodies in gastric glands of the Botiidae and Nemacheilidae/Balitoridae species, consistent with functional acid secretory capacity. In the case of the Botia genus, Verigina [21] reported the presence of gastric glands, although no micrographs were shown and no functional data was provided. In contrast, species in the Cobitidae lacked gastric glands, *atp4a* expression or detectable gastric H^+^/K^+^-ATPase-like immunoreactivity. Thus, we conclude that the morphological absence of gastric glands is strongly correlated with loss of *atp4a* gene expression, a correlation supported by similar observations in other non-gastric lineages [15].

A surprising number of distinct vertebrate lineages show an absence of gastric glands [15], and teleosts in particular have numerous species where gastric glands are absent [11, 12]. Gene loss is a major evolutionary process occurring in animal genomes and may occur rapidly under strong selective pressure [49]. We explored here an intriguing example of stomach presence and absence in the Cypriniformes. In fact, while most families are typically agastric, the Nemacheilidae/Balitoridae sister group in our tree shows a clear gastric phenotype. We conclude that a reinvention of the stomach in Nemacheilidae/Balitoridae was improbable, given that Botiidae have a gastric phenotype. The more parsimonious explanation of our findings is that stomach loss occurred multiple times within the cypriniform lineage, and was retained in the ancestor of the Nemacheilidae/Balitoridae. Thus, we are observing secondary loss in the Cobitidae rather than a reinvention of this complex organ in this group. Furthermore, we show an apparent related loss of the *atp4a* gene, at least in the gastrointestinal tract of the Cobitinae subfamily members. Our study paves the way for more in-depth analyses.

### The controversy over Dollo’s principle

According to Dollo’s principle [50], “Functional or physiological reversal occurs; structural or morphological reversal does not occur” [51]. Therefore, Dollo’s principle predicts that a complex characteristic cannot re-emerge if the loss of the character is followed by the loss of the genetic architecture and developmental mechanisms related to the characteristic [52]. On the other hand, the existence of mutations and recombinations of genes can result in evolutionary reversion even if this characteristic is not regained in the same form [53]. Marshall and co-workers [54] showed that re-acquisition of lost forms is highly improbable after more than 10 MY. Thus, reversals would be statistically improbable if a significant amount of genetic change has accumulated [54]. Some examples of reacquisition of apparently complex features include insects regaining wings [55], lizards reacquiring digits [56], slipper limpets regaining a coiled shell [53], asexual mites recovering sexual reproduction [57], frogs retrieving tadpoles in their life histories [58] and marine snails regaining a feeding larval stage [59], although these are considered analogous structures adapted to their environment. On the other hand, these published phylogenetic exceptions to Dollo’s principle did not document the dates of observed losses and gains, which would have provided insight into potential mechanisms of reacquisition [28]. However, while Weins [58] demonstrated the reacquisition of mandibular teeth in frogs after at least 200 MY, teeth were still present on the upper jaw, and thus the genes and development pathways needed for the development on the lower jaw were preserved. Thus, despite the importance of time lost, the degree of loss can be more relevant for re-emergence of a lost characteristic as is also illustrated for chicken, which are toothless but because the entire pathway for tooth formation remains intact it can be reactivated [28]. This is also recognized as atavism, the re-emergence of ancestral characteristics in individuals. In these cases a lost structure, previously not found in progenitors and lost from several generations to many million years, can appear in a few individuals as in the case of whales born with legs or humans born with a tail) [60]. The accepted explanation for the occurrence of atavism is that the reappearance of the lost structure is possible because of the retention of related genes in the genome for other functions and that as a result of possible mutations, the lost character reappears [60]. In our case of stomach loss, we know the relationship between loss of the gastric phenotype and the loss of related active gastric genes *atp4a* and *atp4b* [15], and maybe pepsinogens [61] that are fundamental to the function of the stomach. However, the presence of gastric genes in the loaches and other stomach-less fishes are still unknown.

### Identification of *atp4a*

Substantial portions of the *atp4a* gene ORF from *C*. *macracanthus*, *B*. *histrionica*, both from the Botiidae family, and *B*. *kweichowensis*, a species belonging to the Nemacheilidae/Balitoridae families, were obtained using PCR. In contrast, various PCR-based attempts to isolate the *atp4a* orthologue from the stomachless Cobitidae family member *M*. *anguillicaudatus* were unsuccessful, consistent with loss of this gene in this species. The alignments for the putative gastric proton pump α-subunit between our three loach sequences and *atp4a* sequences of others species indicate that the loach sequences share a strong degree of identity in the protein functional domains (S3 Fig), and are robustly grouped phylogenetically. The catalytic subunit of the gastric proton pump H^+^/K^+^-ATPase consists of 1034 amino acids with 10 transmembrane segments (TM). Notably, three conserved H^+^/K^+^-ATPase amino acid residues are also conserved in our loach sequences. Cysteine 813, located in the loop between TM5 and TM6, is a cation transporter site that is also a binding site for proton pump inhibitors of the gastric H^+^/K^+^-ATPase [62]. Lysine 791 located in the TM5 segment plays a role in the H^+^/K^+^-ATPase’s outward transport of hydronium ions (H_3_O^+^). Finally, glutamine 820 is involved in K^+^ stimulation of the ATPase activity [63].

The phylogenetic tree of members of the H^+^/K^+^-ATPase α-subunit clade revealed that Atp4A sequences belong to a group markedly different from the Atp1a that encodes the Na^+^/K^+^-ATPase α subunit [8]. A phylogenetic analysis of Atp4a genes of different species indicates that Atlantic stingray (*D*. *sabina*) is the most basally derived vertebrate lineage to have an orthologue of the H^+^/K^+^-ATPase, followed by teleost, amphibian and reptilian representatives, respectively. Indeed, the presence of this gene is correlated with the earliest phylogenetic appearance of gastric acid secretion [32]. The phylogenetic tree also discloses that the three loach Atp4a genes and those of other teleosts, such as stickleback (*G*. *aculeatus*) and Chinese perch (*S*. *chuatsi*), form a strong statistically supported group of fish Atp4a genes.

### Loach systematics

According to Siebert (1987), the Cypriniformes order has two superfamilies: the Cyprinoidea and the Cobitoidea, the latter of which includes the families studied here. Wu and co-workers [64] constructed the first classification of the Cypriniformes based on cladistics argumentation of eight characters [65] and obtained five families. Sawada [66] reorganized the families based on morphological characters using phylogenetic method [67]. Siebert [68] in turn obtained nine families using a phylogenetic approach based on parsimony analysis of 41 characters including primarily cranial osteology. Moreover, on the basis of morphological data, earlier models proposed that the botids were within the same family as the cobitids, and the nemacheilids were within the Balitoridae [13]. However, the phylogeny of the Cobitoidea has been revised more recently using DNA data [22, 24, 69, 70, 71, 72, 73]. Through the use of the mitochondrial DNA genes cytochrome b and control region, and the nuclear recombination activating gene 1 (RAG-1), the Nemacheilidae and Balitoridae have been reassigned as separate sister families, and the Botiidae and Cobitidae are now recognised as different families. These DNA data converge to identify seven Cobitoidea families, which we used as the foundation for our study [22]. However, the higher level sister group relationships among these families remain unresolved, possibly owing to the limited number of genes or taxa analyzed. According to Swofford and co-workers [74], a phylogenetic reconstruction should follow precise analytical methods, appropriate and sufficient morphology, and molecular and behavioural data, in addition to appropriate selection of taxa. Unfortunately, incomplete understanding of the families, subfamilies and other groups still causes uncertainty [72]. However, there is an ongoing international effort directed at investigating the systematics of the Cypriniformes, so more information about their genealogical relationships is certain to emerge.

The controversies surrounding the morphological and phylogenetic classification of loaches justify further study of diverse gastrointestinal tract characteristics as a novel approach to clarification of loach systematics. Our study has established the stomach phenotype with the presence of gastric glands and *atp4a*/Atp4a expression in members of the Nemacheilidae, Balitoridae and Botiidae families, and absence of both characteristics in the Cobitidae. Yet to be investigated is the small Cobitoidea family Vaillantellidae that is ancestral to the agastric Cobitidae family, and gastric families Botiidae and Nemacheilidae/Balitoridae. We would predict that the Vaillantellidae are a gastric family. Determination of the extent of the stomach loss in the Cobitidae, of which we have studied only two of 18 genera, would be most informative.

## Supporting Information

S1 Fig Alignment of the oligopeptide used to generate the NAK121 and αR1 antibodies with the corresponding amino acid sequences from *Xenopus tropicalus* Atp1a1, Atp4a, and Atp12a. (DOCX)

S2 Fig Immunohistochemical localization of HKα1 using the C2 antibody in the stomach of the outgroup (a) Siluriformes channel catfish (*Ictalurus punctatus*) and (b) Characiformes Mexican tetra (*Astyanax mexicanus*). Images are overlaid with DAPI and DIC. Scale bar 100μm.
(DOCX)

S3 Fig Alignment of a partial sequence of gastric HKα1 (ATP4A) in a number of vertebrates. ATP1A1 and ATP12A in *H*. *sapiens* and *X*. *tropicalis* are shown for comparison. Red boxes: Potassium binding by lysine (K) protonated at pH<3 and Glutamate (E) with two sites. Blue box: Cysteine (C) is inhibited by omeprazole (SCH28080).
(DOCX)

S1 Table Nucleotide sequence of primers for β-actin and *atp4a* degenerate and consensus primers. (DOCX)

S2 Table Nucleotide sequences of the different sets of primers used to test for the presence of *atp4a* sequence in *C*. *macracanthus*, *B*. *histrionica*, *B*. *kweichowensis*, and *M*. *anguillicaudatus*. (DOCX)
