# Supplementary material for: The Gastric Phenotype in the Cypriniform Loaches: A Case of Reinvention?
Source: PLoS One. 2016 Oct 26;11(10):e0163696. doi: 10.1371/journal.pone.0163696 (PMC5082673; doi:10.1371/journal.pone.0163696)
Supplement: S1 Fig — (DOCX) [file pone.0163696.s001.docx]

**Supplemental material**

NAK121 peptide VTGVEEGRLIFDNLKKS

Xenopus Atp1a VTGVEEGRLIFDNLKKS 17/17

Xenopus Atp12a VTGVEEGRLIFDNLKKS 17/17

Xenopus Atp4a VTGVEQGRLIFDNLKKS 16/17

**S1 Fig.** Alignment of the Ura et al. (996) oligopeptide used to generate the NAK121 and αR1 antibodies with the corresponding amino acid sequences from *Xenopus tropicalus* Atp1a1, Atp4a, and Atp12a.
