# Supplementary material for: The Gastric Phenotype in the Cypriniform Loaches: A Case of Reinvention?
Source: PLoS One. 2016 Oct 26;11(10):e0163696. doi: 10.1371/journal.pone.0163696 (PMC5082673; doi:10.1371/journal.pone.0163696)
Supplement: S2 Fig — Images are overlaid with DAPI and DIC. Scale bar 100μm. (DOCX) [file pone.0163696.s002.docx]

**Supplemental material**


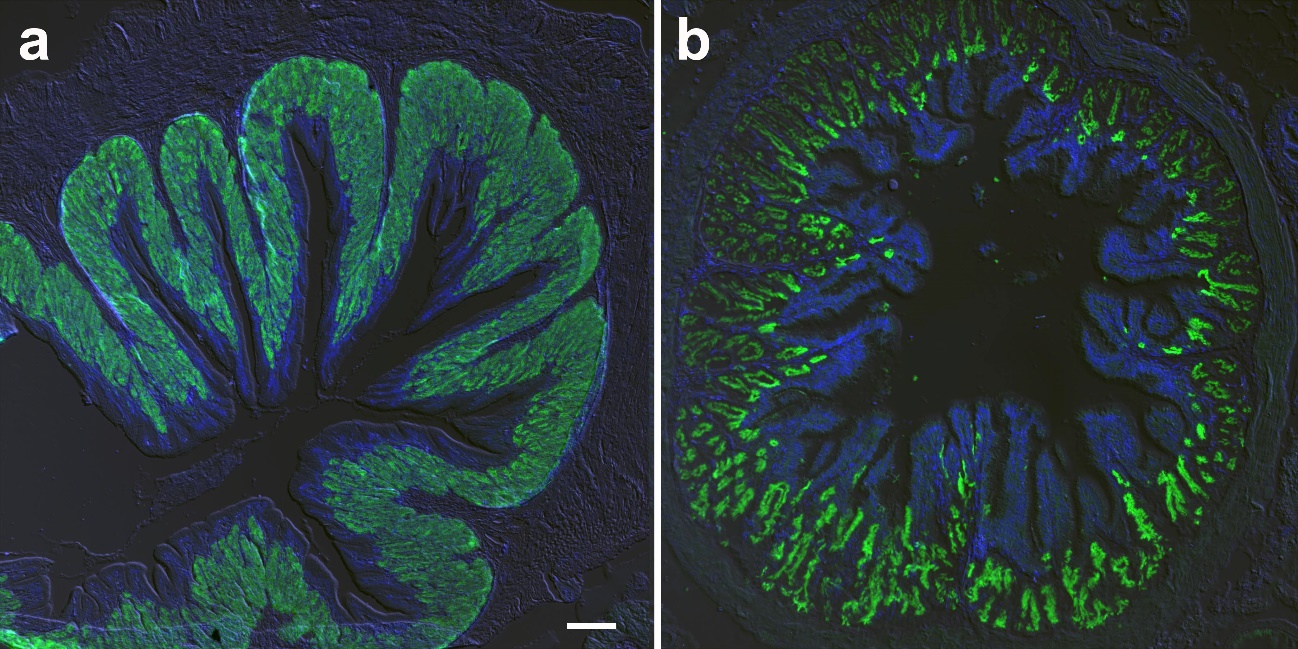


**S2 Fig.** Immunohistochemical localization of HKα1 using the C2 antibody in the stomach of the outgroup (a) Siluriformes channel catfish (*Ictalurus punctatus*) and (b) Characiformes Mexican tetra (*Astyanax mexicanus*). Images are overlaid with DAPI and DIC. Scale bar 100µm.
