# Supplementary material for: The Gastric Phenotype in the Cypriniform Loaches: A Case of Reinvention?
Source: PLoS One. 2016 Oct 26;11(10):e0163696. doi: 10.1371/journal.pone.0163696 (PMC5082673; doi:10.1371/journal.pone.0163696)
Supplement: S3 Fig — ATP1A1 and ATP12A in H. sapiens and X. tropicalis are shown for comparison. Red boxes: Potassium binding by lysine (K) protonated at pH<3 and Glutamate (E) with two sites. Blue box: Cysteine (C) is inhibited by omeprazole (SCH28080). (DOCX) [file pone.0163696.s003.docx]

**Supplemental material**


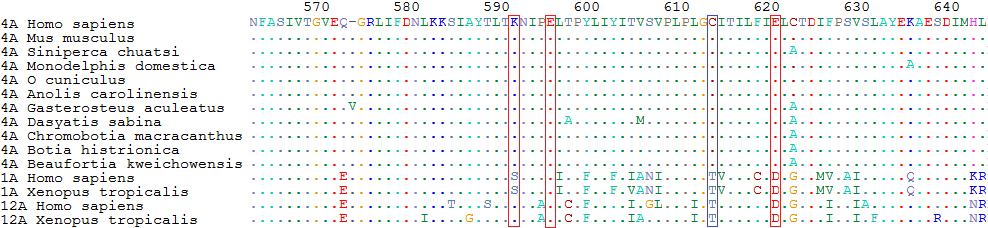


**770 780 790 800 810 820 830 840**

**S3 Fig.** Characteristic amino acid residues for gastric HKα1 (ATP4A). Red box: Potassium binding by lysine (K) protonated at pH<3 and Glutamate (E) with two sites. Blue box: Cysteine (C) is inhibited by omeprazole (SCH28080). Amino acid positions relative to *H. sapiens* ATP4A.
