# Supplementary material for: The Gastric Phenotype in the Cypriniform Loaches: A Case of Reinvention?
Source: PLoS One. 2016 Oct 26;11(10):e0163696. doi: 10.1371/journal.pone.0163696 (PMC5082673; doi:10.1371/journal.pone.0163696)
Supplement: S1 Table — (DOCX) [file pone.0163696.s004.docx]

**Supplemental material**

**S1 Table.** Nucleotide sequence of primers for β-actin (*actb*) and *atp4a* degenerate and consensus primers.

| **Gene** | **Primer Name** | **Sequence (5’-3’)** | **Observations** |
| --- | --- | --- | --- |
| ***actb*** | SbrAct-F* | GGCCGCGACCTACAGACTAC | * Santos et al., 1997 |
|  | SbrAct-R* | ACCGAGGAAGGATGGCTGGAA |  |
| ***atp4a*** | ATP4A F1d** | GAYGARCARTGGAARGARGC | **Choe et al. 2004 |
|  | ATP4A R1d** | GGRAACCANCCYTCYTGNGCC |  |
|  | ATP4A_F318d** | NCTNCARTGYCTNATGTGGGT |  |
|  | DF4A_R1742 | GTGGGGAAATTCATCTCAT |  |
