# Supplementary material for: The Gastric Phenotype in the Cypriniform Loaches: A Case of Reinvention?
Source: PLoS One. 2016 Oct 26;11(10):e0163696. doi: 10.1371/journal.pone.0163696 (PMC5082673; doi:10.1371/journal.pone.0163696)
Supplement: S2 Table — (DOCX) [file pone.0163696.s005.docx]

**Supplemental material**

**S2 Table**. Nucleotide sequences of the different set of primers used to test the presence of *atp4a* sequence in *C. macracanthus*, *B. histrionica*, *B. kweichowensis, M.anguillicaudatus*

| **Name** | **Primers** |
| --- | --- |
| **ZBLoach4A_RACE_R2** | CGAAAGACTGGATGGCACCTATCTG |
| **ZBLoach4A_RACE_R1** | TGAAGAGTCCCTGCTGGAAGAGAGA |
| **ZBLoach4A_RACE_F2** | GGGACTCTTCAGGAACAAGGTGCTT |
| **ZBLoach4A_RACE_F1** | CTGCAGGACAGTTACGGACAGGAGT |
| **ZBloach4A2RACE_R2** | AGGGAATAATGTCTGTGCCCAGCTC |
| **ZBloach4A2RACE_R1** | GTAGCCTTGTTCAGCCATCACGGTA |
| **ZBloach4A2RACE_F2** | CAAGGCTACCTTCCTGGAACACTGA |
| **ZBloach4A2RACE_F1** | GAGGAAGGTCGTCTGATCTTCGACA |
|  |  |
| **degATP12A-R2** | TTCGCTCCGGCgcnccyttcat |
| **degATP12A-F2** | CAAGCGGATGGCAAAGaaraaytgyyt |
| **degATP12A-R1** | CGCGATGGAAACAaaccartaytg |
| **degATP12A-F1** | CGCCGACATGATACTACTAGATgayaayttygc |
|  |  |
| **HKA-a1-rev2** | AACAGCTGGAAGACGATGG |
| **HKA-a1-rev1** | CCTGTTGGAAGATGGACAG |
| **HKA-a1-fwd3** | TGTCTCTGGCCTATGAGAAG |
| **HKA-a1-fwd2** | AGAAACTGATTATCGTGGAGA |
| **HKA-a1-fwd1** | GCATCATCTCAGAAGGCAGT |
|  |  |
| **Choe4A_R1d** | GGR AAC CAN CCY TCY TGN GCC |
| **Choe4A_F1d** | GAY GAR CAR TGG AAR GAR GC |
